# Solitary fibrous tumor of the trachea: a case report

**DOI:** 10.1007/s11748-019-01274-5

**Published:** 2019-12-17

**Authors:** Masahiro Kitada, Shunsuke Yasuda, Masahiro Abe, Nana Yoshida, Satoshi Okazaki, Kei Ishibashi

**Affiliations:** Department of Respiratory Center, Asahikawa Medical University, Midorigaoka-Higashi 2-1-1-1, Asahikawa, Hokkaido 078-8510 Japan

**Keywords:** Tracheal tumor, Solitary fibrous tumor, Tracheal resection

## Abstract

We experienced a surgical case of a rare primary tracheal tumor. A 77-year-old woman visited a local clinic with chief complaints of coughing, wheezing, and discomfort in the throat. Computed tomography revealed a mass measuring approximately 1.5 cm in the mediastinal trachea, extending from the membranous portion of the trachea to the esophagus. Bronchofibroscopy showed a flat, smooth-surfaced, round mass arising from the membranous portion. Surgery was performed because of the possibility of airway obstruction and suffocation. Sleeve resection of five tracheal rings was performed via median sternotomy and interrupted suture was performed using 3-0 absorbable suture material. The postoperative course was favorable and there has been no evidence of recurrence. The pathological diagnosis was solitary fibrous tumor. A primary solitary fibrous tumor of the trachea is extremely rare. Here, we report this disease with a literature review.

## Introduction

Primary tracheal tumors are relatively rare and at least 90% of these tumors are malignant. Among them, solitary fibrous tumors (SFTs) are extremely rare tumors. Tracheal tumors can cause airway obstruction by tumor growth; therefore, early treatment is essential. We experienced a surgical case of an SFT presenting with symptoms of coughing and wheezing.

## Case

**Fig. 1:**
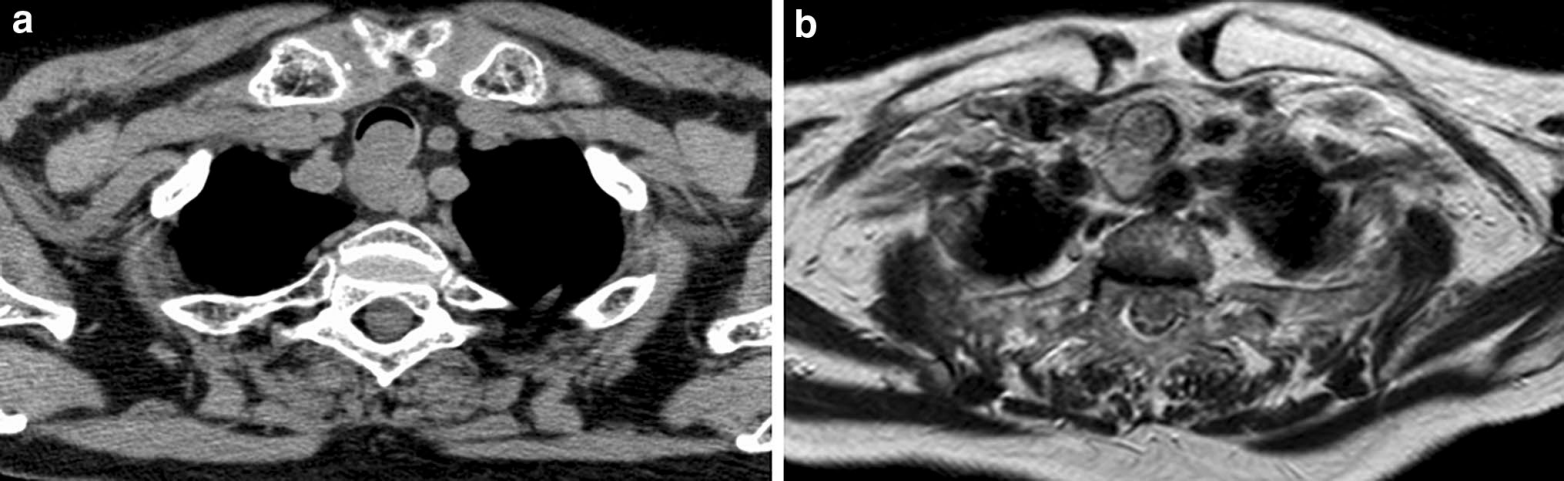
Computed tomography (CT) and magnetic resonance imaging (MRI) findings revealed a 1.5 cm large border and a smooth surface nodule shadow on the posterior wall of the thoracic trachea approximately 5 cm below the glottis. Mass formation with a major axis of 1.0 cm in diameter extending from the trachea to the esophagus was observed. Based on MRI findings, the border with the surrounding organs was clear, and it was diagnosed as primary tracheal tumor invasion (CT: **a**, MRI: **b**)

**Fig. 2:**
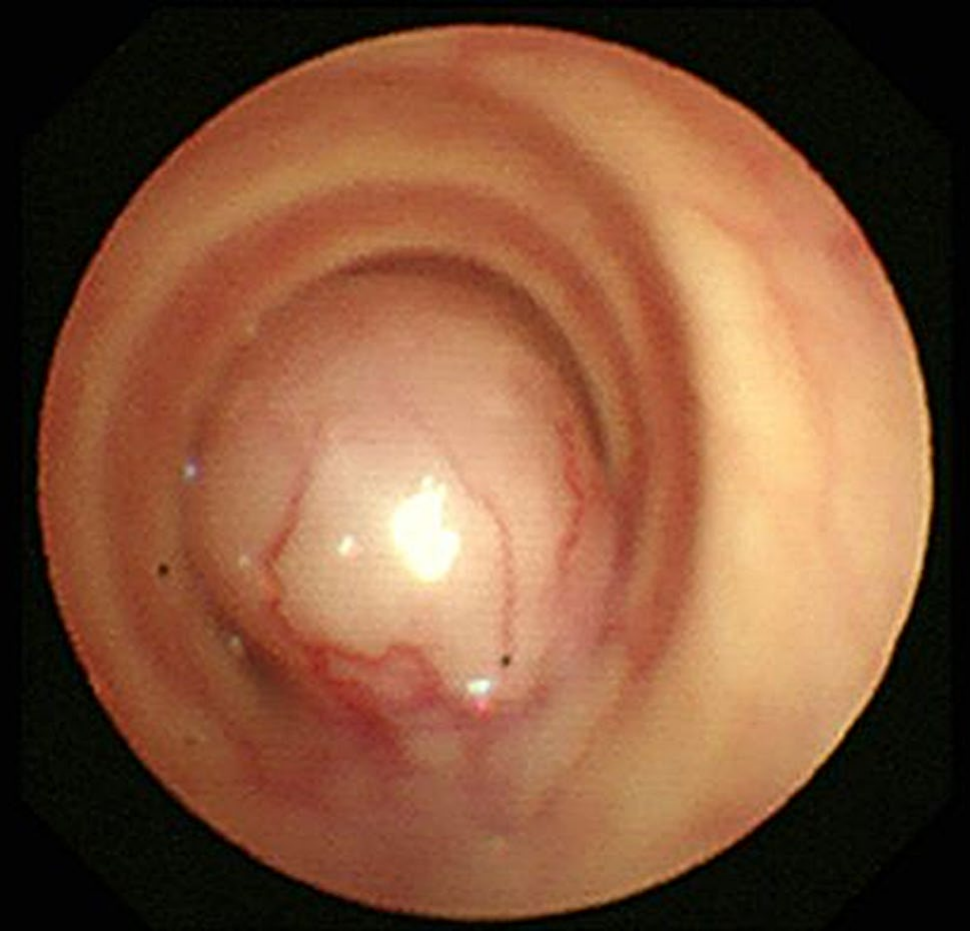
Bronchoscopy showed a smooth-surfaced submucosal tumor with abundant neovessels, which accounted for 80% of the cross-sectional area of the trachea

**Fig. 3:**
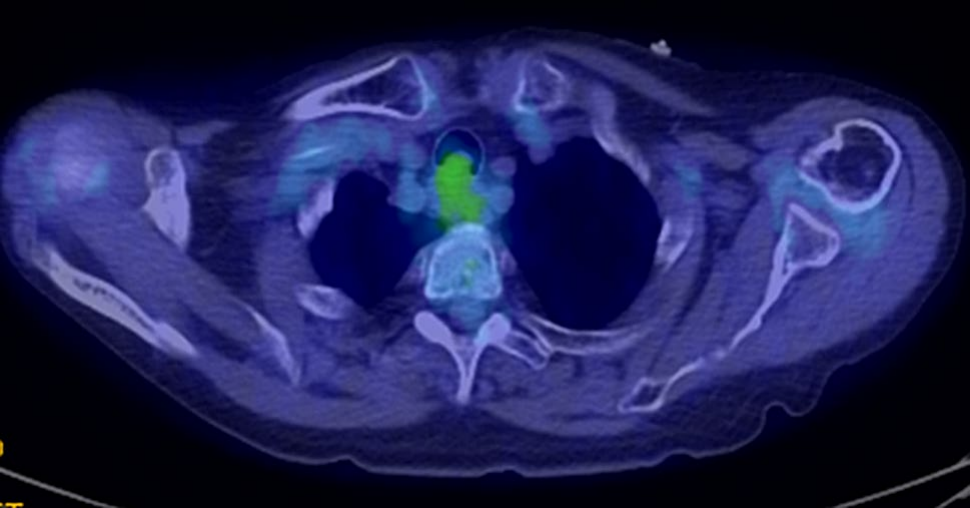
FDG-PET showed accumulation, with a maximum standardized uptake value of 2.9 for the lesion, and the possibility of malignancy could not be excluded

**Fig. 4:**
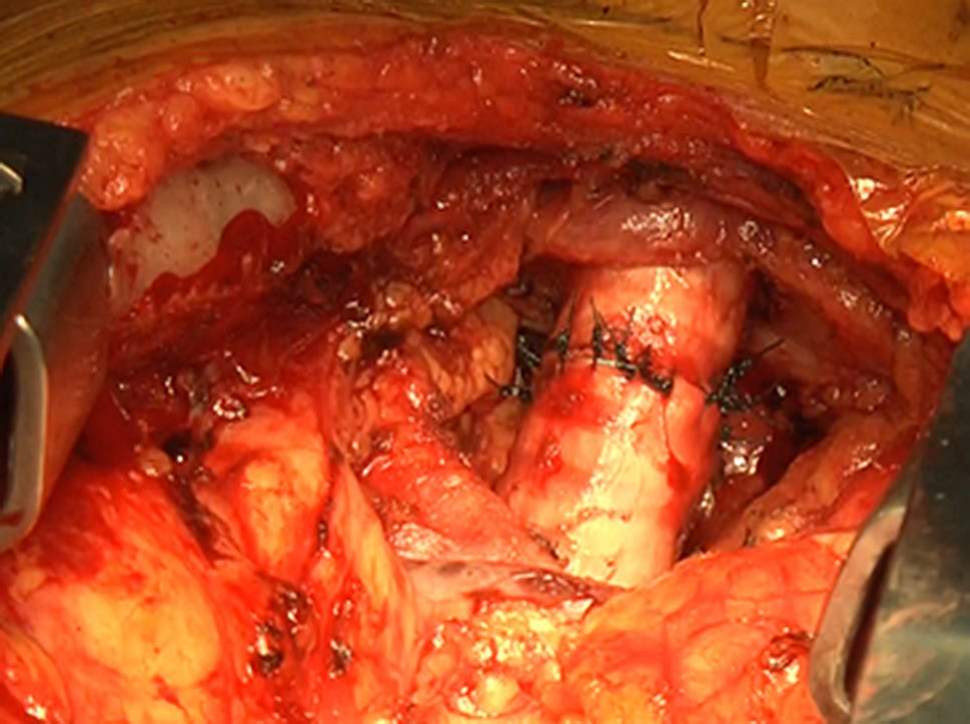
End-to-end anastomosis of the trachea was performed using 3-0 monofilament synthetic absorbable suture material. A continuous suture was initiated in the membranous portion. The orally inserted tracheal tube was then advanced to the distal portion in order to secure the airway, and interrupted suture of the portion of the tracheal cartilage was performed

**Fig. 5:**
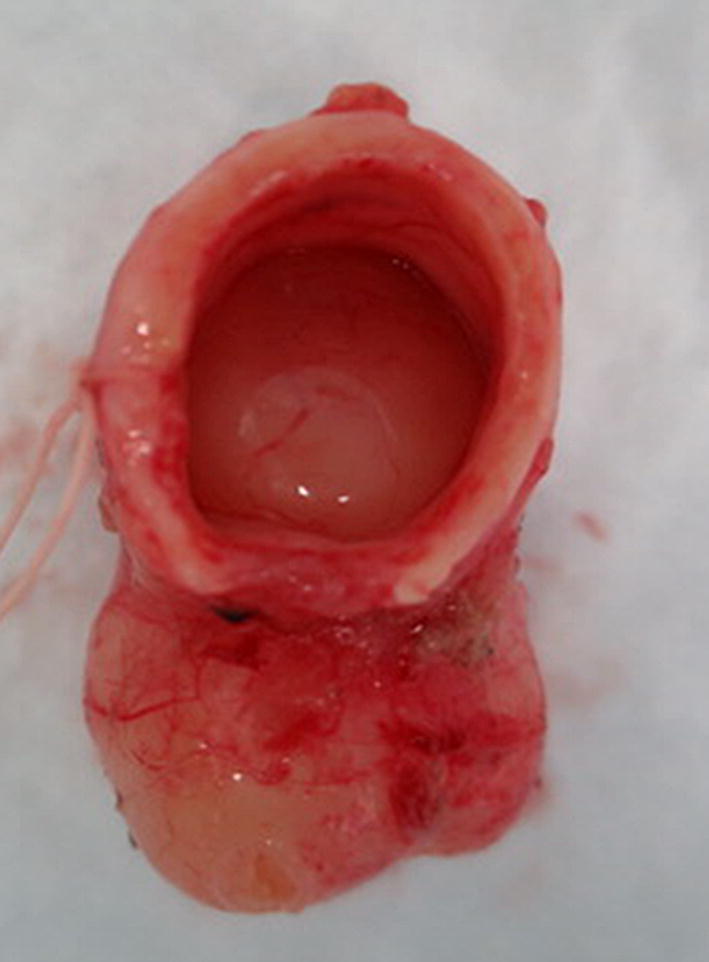
Excised specimen: The resected specimen showed a white–pink tumor (maximum diameter of 23 mm) protruding into the tracheal lumen and the membranous portion

**Fig. 6:**
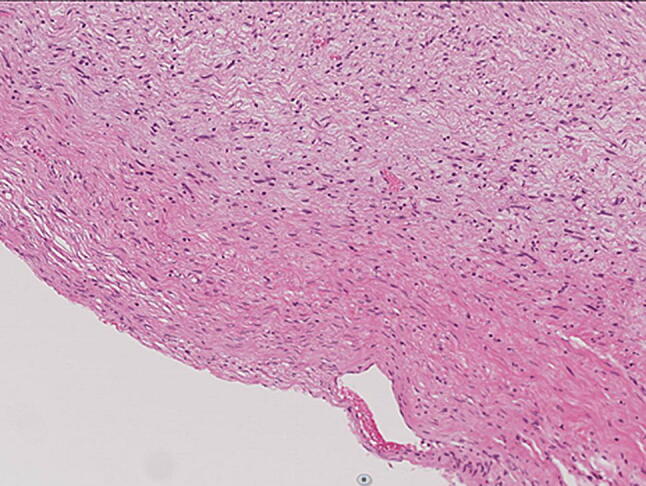
Pathological examination showed a mass of hyperplastic atypical cells with spindle-shaped nuclei arranged in bundles running in various directions in the submucosa (HE ×200)

**Fig. 7:**
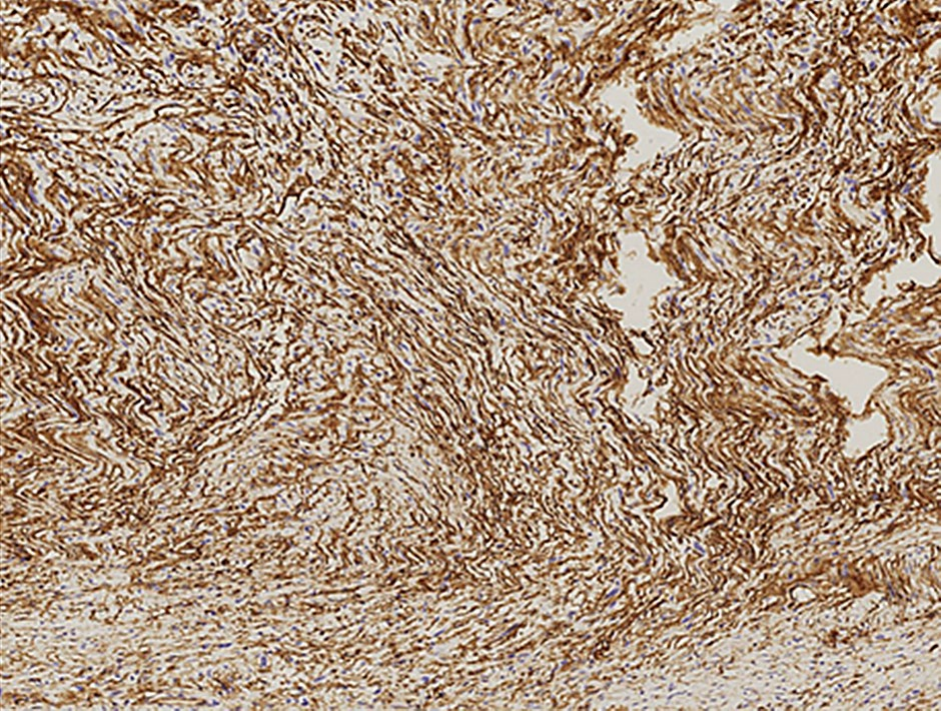
Immunostaining, the tumor was positive for vimentin (×200)

A 77-year-old woman visited a local clinic because of persistent cough for the past 6 months. A tracheal tumor was suspected on imaging and she was referred to us for further evaluation and treatment. Her medical history included hypertension and atrial fibrillation, for which she was treated with medications. Her height was 156 cm and weight was 56 kg. Respiratory noise was audible on respiration. There was no enlargement of surface lymph nodes. Additionally, there were no abnormalities in blood biochemistry and tumor marker levels were normal. Radiography showed a mass in the trachea. Computed tomography (CT) and magnetic resonance imaging (MRI) findings revealed a 1.5 cm large border and a smooth surface nodule shadow on the posterior wall of the thoracic trachea approximately 5 cm below the glottis. Mass formation with a major axis of 1.0 cm in diameter extending from the trachea to the esophagus was observed. Based on MRI findings, the border with the surrounding organs was clear and it was diagnosed as primary tracheal tumor invasion (Fig. 1a, b) Furthermore, bronchoscopy showed a smooth-surfaced submucosal tumor with abundant neovessels, which accounted for 80% of the cross-sectional area of the trachea (Fig. 2). Biopsy was not performed, because there was a strong possibility of airway obstruction due to bleeding. Fluorodeoxyglucose-positron emission tomography (FDG-PET) showed accumulation with a maximum standardized uptake value of 2.9 for the lesion and the possibility of malignancy could not be excluded (Fig. 3). The patient was not in a state of respiratory distress and tumor resection using a bronchoscope was at risk of bleeding, we decided to remove the tumor by tracheal sleeve resection, because the patient had symptoms and was at risk of airway obstruction. Regarding anesthesia, the percutaneous cardiopulmonary support (PCPS) was put on standby in consideration of the possibility of suffocation due to intubation difficulties and bleeding during intubation. There were no problems during anesthesia except for the operation of the intubation tube. Surgery was performed via median sternotomy. The thymus and surrounding structures were separated to expose the trachea, and then five tracheal rings, including the tumor, were removed by sleeve resection. Separation from the esophagus was relatively easy. After resection of the trachea, the airway was secured by intubation. End-to-end anastomosis of the trachea was performed using 3-0 monofilament synthetic absorbable suture material. A continuous suture was initiated in the membranous portion. The orally inserted tracheal tube was then advanced to the distal portion to secure the airway and interrupted suture of the portion of the tracheal cartilage was performed. The anastomosis site was covered with the thymus (Fig. 4). Five tracheal rings were excised for excision of one or more tracheal rings above and below the tumor. Based on the results of intraoperative pathology, it was judged that there was no tendency for tumor infiltration and additional resection was not performed. The postoperative course was favorable. There has been no recurrence for 2 years after surgery. The resected specimen showed a white–pink tumor (maximum diameter of 23 mm) protruding into the tracheal lumen and the membranous portion (Fig. 5). Pathological examination showed a mass of hyperplastic atypical cells with spindle-shaped nuclei arranged in bundles running in various directions in the submucosa. On immunostaining, the tumor was positive for vimentin, CD34, and Bcl-2, and negative for α-smooth muscle actin (α-SMA). Based on these findings, the tumor was diagnosed as an SFT (Fig. 6, 7). There were no malignant findings and the resected stump was negative. The postoperative course was favorable and there has been no evidence of recurrence for 2 years after the surgery.

## Discussion

Primary tracheal tumors are relatively rare. At least 90% of these tumors are progressive malignant tumors such as adenoid cystic carcinoma and squamous cell carcinoma [1, 2]. On the other hand, less than 10% of these tumors are benign tumors, including relatively frequent squamous papilloma [3] and other tumors such as inflammatory myofibroblastic tumors [4] and leiomyoma [5], and SFTs are extremely rare.

An SFT was first described in 1931 by Klemperer as an intermediate malignant tumor arising in the pleura [6]. According to the new WHO classification [7], an SFT is an intermediate malignant mesenchymal tumor classified as a fibroblastic/myofibroblastic tumor. Initially, it was believed that most SFTs have a pleural origin. However, since the 1990s, there has been an increasing number of reports on SFTs arising from a variety of extrapleural sites, including the peritoneum, mediastinum, lungs, thyroid, rhinopharynx, orbit, lacrimal sac, vagina, scrotum, and soft tissues, but SFTs arising from the tracheal bronchus are extremely rare, and only few cases have been reported [8, 9]. In addition, there are reported cases of SFTs presenting with endocrine symptoms such as non-islet cell tumor hypoglycemia [10]. The localization diagnosis of an SFT can be made by imaging, but its differential diagnosis from other soft tissue tumors is difficult because of the absence of specific findings associated with an SFT on imaging studies, including FDG-PET. Therefore, a definite diagnosis is only achieved after histopathological examination. Pathologically, spindle-shaped cells are arranged in bundles running in various directions and they proliferate in the interstitium containing collagen fibers. On immunostaining, the tumor is diffusely and strongly positive for CD34, an antigen in fibroblastic cells, as well as vimentin and Bcl-2. On the other hand, it is negative for S-100 and α-SMA, which are involved in the differentiation of neurons and muscles, indicating that this tumor originates from mesenchymal cells just below the mesothelium [11].

Tracheal tumors can cause airway obstruction by tumor growth and they might cause suffocation, depending on their progression; therefore, early treatment is essential. A bronchoscopic biopsy is needed for a definite diagnosis, but intraoperative rapid diagnosis is recommended when bronchoscopy has a risk of airway obstruction due to bleeding. In the present case, surgery was performed without preoperative tumor biopsy, because the size of the tumor was large, accounting for the majority of the cross-sectional area of the trachea and the excision margin of the bronchus and tumor was the length of one tracheal ring.

The treatment principle of primary tracheal tumors is tumor removal via tracheal segmental resection, because there are risks of suffocation and bleeding associated with tumor growth and perforation of the membranous portion. A relatively small pedunculated mass might be successfully removed using a high-frequency snare and laser [12]; however, in the present case, surgical resection was needed, because the tumor extended from the membranous portion of the trachea to the esophagus. Surgery for tracheal tumors mainly involves sleeve resection of the trachea and direct anastomosis and resection of seven to eight tracheal rings has been reported to be safe [13]. In cases requiring resection of more than eight tracheal rings, additional treatments, such as hilar mobilization and pulmonary ligament dissection, might be needed. Our patient did not receive additional treatments, such as tracheal mobilization, because she was intraoperatively diagnosed with an SFT and did not require massive resection that is usually needed in patients with malignancy. With regard to suture, non-absorbable polypropylene suture material is used in many institutions.

## Conclusion

We reported a rare case of a primary SFT of the trachea treated with tracheal sleeve resection and anastomosis.

## Availability of data and materials

All data generated or analyzed during this study are included in this published article.

## Abbreviations

SFT: Solitary fibrous tumor
CT: Computed tomography
MRI: Magnetic resonance imaging
FDG-PET: Fluorodeoxyglucose-positron emission tomography
PCPS: Percutaneous cardiopulmonary support
